# Clinical Reports

**Published:** 1838

**Authors:** 


### WESTERN JOURNAL

OF THE

### MEDICAL AND PHYSICAL SCIENCES.

**For April, May, and June, 1838.**

### ESSAYS AND CASES.

### Art I.—Clinical Reports.

*Introductory Remarks.*

It is our intention henceforth, to group all the clinical cases, which may be published in the Journal, under one head, the first of each number. There will be an advantage to our correspondents in this, in as much, as a case, however important, when thrown in among essays, dissertations and histories of epidemics, is liable to be overlooked. In the present number our own clinical reports, are postponed to make room for those of correspondents, but they will appear in October. Convinced that all our readers, meet very frequently with cases, which are worthy of being reported, we respectfully solicit their contribution towards our *Western clinique*, which may thus, we flatter ourselves, be rendered interesting, both at home and abroad.. If we occasionally take the liberty of abridging what is sent to us (looking faithfully to the preservation and presentation of the main facts) we hope it will not be considered an unwarrantable liberty. We trust, also, that we shall give no offence, by saying that most of the reports sent us, are not drawn up with the care that might easily be practiced. The manual execution is often so bad as to obscure the meaning of the author; and in giving the recipies, the names of the medicines are frequently too much abreviated, and the quantities too uncertain, while the union of common and scientific—of old and new—of exploded and unexploded—pharmaceutic terms occasionally borders on the ludicrous. Many physicians seem to consider the reporting of cases, as a business that may be turned off their hands with less labor than an essay; but we are of different opinion. The world is overwhelmed with reports of cases, three-fourths of which are never read by any body, for the very substantial reason that they are unreadable; or if any one has the courage to travel through them, he finds confusion and prolixity; want of discrimination between the essential and unessential; redundance on the one hand and meagerness on the other; the triumphant establishment of a common-placeism, which had been re-established and re-affirmed annually since the year in which Hippocrates graduated; finally, a jumble of the past and present tenses of the verbs, which makes the reader uncertain whether the patient was or is under treatment. In truth we believe, that the literature of clinical cases, is at once the most neglected and the most difficult in the profession; that this remark is not applicable to the West, or even the United States, merely, and that its better cultivation would contribute largely to the improvement of medical science; but as it is uncertain whether any body will think with us, we shall say no more, lest we be charged with the affectation of oddity. D.

---

Obliteration of the Vagina of ten months standing, cured by the knife. By Dr. Robert Robson, *of New Harmony, Ia.*

In May 1836 I was called to visit Mrs. H., living near New Harmony, Ia., and informed by her parents, that in the year 1835 while in a state of pregnancy, she had a severe attack of remittent fever, which terminated in abortion; after which, she slowly recovered strength for some weeks; when she suddenly became afflicted with uterine pains, affecting the loins, hips and lower part of the abdomen. Frequent applications were then made to her physician, without, however, making him acquainted with her real situation, and medicines were administered which only tended to increase the violence of her pains. In this way she continued for some months. I found her pale and reduced to a state bordering on marasmus, with restless nights, loss of appetite, &c. Her uterine pains were strong, producing the appearance of a firm tumor half an inch within the vulva, which yielded to pressure. On further and more minute examination, I found the vagina at that point completely obliterated. The knife seemed the only resource, and agreeably to the request of the patient and her friends, I employed it. I made a crucial incision in the direction of the os tincæ, when an immense quantity of black putrid blood, of the consistence of molasses, was slowly, but regularly discharged, mixed with lumps or flakes of putrid matter which stopped up the orifice, and had to be removed in order to continue the flow.

A strong glass tube of a conical form, open at both ends, and sheathed in one of gum elastic, was then dipped in olive oil and the small end introduced into the newly made orifice, which answered the double purpose of dilating the parts and admitting the accumulations of the uterus to pass off. A dose of ol. Ricini was then administered and the patient put to bed, when she enjoyed for the first time in many months, several hours of uninterrupted sleep. I continued to visit her, and found the bad symptoms gradually subsiding, and by the assistance of tonics conjoined with laxatives, she was finally restored to perfect health.

---

Facial Neuralgia cured by the Atropa Belladona.—*By the same.*

Mrs. B. of New Harmony, of delicate constitution, subject to chills and fevers, went to bed on the night of 22d of January 1834, quite well. She awoke about sun-rise with a strange creeping and tingling sensation in the left temple, eye-ball, and adjacent parts, which very soon increased to an intolerable burning, and at length to an acute pain. The paroxysm lasted with seemingly increased violence, until the turn of the day, when she gradually experienced a remission of pain, and by evening was quite easy. I was called to see her at nine o'clock in the morning. *Symptoms.* In addition to the symptoms just enumerated, she had a small pulse, skin dry and rather hot, tongue coated, bowels open, with pain about the epigastric and left hypochondriac region. Believing the case to be connected with congestion and derangement of the digestive organs, I prescribed venesection to 20 ounces; and calomel and opium to be followed by aperients, to the seat of the pain a turpentine liniment.

23d. Eight o'clock A. M.—Medicine operated well, has had three or four bilious evacuations; but the pain had returned and was intense, affecting the infra orbital nerve; complains much, thinks her eye will burst, pains in the left hypochondriac region better, but it still continues tense and sore on pressure. *Prescription*—calomel, followed by ol. Ricini—a blister to the left temple extended over the forehead, and repeated doses of the sedative solution of opium during the paroxysm.

24th. Eight o'clock A. M.—Thinks herself getting worse, pain most excruciating, complains of loss of vision, pupil dilated, and a partial paralysis of the upper eye-lid, bowels open, and pain in the left hypochondriac region, much relieved. *Prescription*—Sulph. Quin. in large doses every two hours during the intermission, and the Sulph. Quin. on cerate applied over the raw surface, occasioned by the blisters.

25th. Nine o'clock A. M.—Pain excessively severe and radiateing across the temple and up the forehead, into the right orbit; declares she cannot live without some immediate relief, pupil much dilated, but can see at short distances, parts above the orbit swollen, &c. Seeing that the pains still continued without the slightest abatement from the treatment heretofore pursued, I resolved to try the Atropa Belladona, when 20 grains were dissolved in acetic acid, ℥j. and rubbed for a few minutes over the superciliary arch, a linen cloth was then moistened and applied over the os frontis, eye-ball, and left temple, when an almost instantaneous cessation of pain was experienced which continued until the following morning, when some slight pain was again felt and the Atropa Belladona again applied, when she was entirely relieved and has had no subsequent attack.

### Editorial Remark.

This was a case of miasmatic Neuralgia—disguised intermittent—many of which occur in the West. We have not tried the local applications of the extract of Belladona, having been satisfied with the internal administration of the Sulphate of Quinine, which we are disposed to think was in reality the chief remedy in this case. D.

---

Osteo-Sarcoma and Excision of a large portion of the Lower jaw. By Dr. J. Wort, *of Jackson county, Indiana*, March 1838.

The subject of this case is Mr. Gerardus Ryker, of Jefferson county Ia., aged 71 years. He is a man of good habits, and has always enjoyed good health, except an attack of white swelling in both legs, about the time of puberty.

About two years ago, he received a severe contusion on the inferior maxillary bone, by the falling of his horse on the ice, which he thought at the time, had fractured the jaw. In a few days he recovered from this injury, and had forgotten the circumstance till about five months afterwards, when he was seized with most excruciating pain, attended with violent inflammation and swelling of the jaw. This subsided after a few days, by the use of the topical vapor bath, leaving a knot about the size of a small bullet, on the exterior surface of the right side, midway between the centre of the chin and the angle of the bone, apparently immovable. This continued to increase gradually, with occasional paroxysms of severe inflammation, and excruciating pain, till about six months since, when the pain became continual and lacinating, frequently producing severe spasms, with continued fever and constipated bowels. The tumor meanwhile rapidly increased; he had consulted many surgeons, but they all considered his case as hopeless.

In January last, Dr. W. Davidson, of Madison, a graduate of Edinburgh, and a gentleman of fine medical acquirements, was attending to his case. I was written to by the Doctor for my opinion and advice, and shortly afterwards was called on to see the patient. I visited him on the 19th January, they having put off calling on me for some time after receiving my answer to Doctor Davidson's letter, believing him to be in a dying condition.

I found him very feeble, emaciated, and haggard in his appearance, but he became much animated, and joyful on my arrival; so great was his anxiety, and strong his hopes that I would operate on him, and give him the only remaining chance of life, or terminate his protracted and unsupportable sufferings.

The tumor was an enlargement of the inferior maxilla, and a change or softening of its substance; the enlargement extended from the neck of the condyloid process to the centre of the chin, on the one hand, and up to the zygomatic process of the malar bone, on the other. From the right ear it reached to within one inch to the left of the trachea, projecting outwardly nearly two inches, and inwardly under the tongue to near the centre of that organ. Below, it involved the throat, pushing the hyoides three-fourths of an inch down to the left side, and compressing the external carotid artery to near its union with the internal carotid. It interrupted his speech, deglutition, and respiration very much, so that at times he was threatened with immediate suffocation.

I waited till next morning to deliberate on the matter, and from his strong entreaty and perfect resignation to the result of an operation, I concluded to perform it the next day. I therefore called on Doctors Davidson and Hall, of Madison, Ia., to assist in the operation. Having premised a gentle aperient the night before, andgiven him a grain of morphine half an hour previous to the operation, he was laid on a table with his head near a window, with a mattrass under him. I stood on his right side, and one assistant on each side of the table, provided with nothing more than an ordinary pocket case of instruments, sponge, water, &c. I commenced the incision by cutting down to the solid tumor, extending from the tragus of the ear to within one inch of the centre of the chin, being as far as the tumor reached in that direction. Another incision decussated the first at right angles, each being seven inches long, being just the diameter of the tumor. I then carefully dissected the skin from over the tumor, in four angular flaps, cutting as close as possible to the skin, leaving all muscles &c., with the flaps. I then began to dissect the tumor from above, near the malar bone, keeping close to the tumor. Inwardly, there was nothing but the lining membrane of the cheek left, as the teeth were all gone. I next cut through the jaw bone at the chin, and found it (as I hadanticipated) soft and brittle. I next dissected out the process of the tumor which extended under the left side of the throat and over the trachea. I had frequently to stop to restrain hemorrhage. The small vessels were generally secured by tortion, and the wound cleansed of blood, and the discharge from minute vessels restrained by the application of a solution of kreosote, one part to fifty of water, (an application which I use in many surgical cases to advantage.) I now dissected the tumor from the carotid artery and jugular vein; the principal part of the parotid gland being involved in the tumor, it had to be removed. Dissecting round and leaving the principal branch of the internal maxillary artery and the temporal artery unhurt, I sought for the inferior maxilliary artery and vein, to take them up before they were cut; the vein was anterior to the artery and very large, being about the size of a turkey quill. I put a ligature round it close to the tumor and cut it, to give room to search for the maxillary artery, but as soon as I cut it, the blood regurgitated from the jugular vein as thick as a quill, and the patient being much exhausted, fainted. We then had to restrain the hemorrhage with a sponge, wetted in a solution of kreosote, till he was resussitated by proper stimulants. This vein retracted and could not be secured till the tumor was removed, or part of it at least. I then at one stroke of the scapel, took off so much of the tumor as was dissected loose, and cut through the maxillary artery, which had been drawn so far out of its proper place by the tumor, that it retracted as soon as bisected, and was so contiguous to the carotid, and such a profusion of blood, that securing these vessels was a most difficult part of the operation. These secured, I proceeded to dissect out the balance of the tumor with the jaw bone.

I cut through the bone again at the neck of the condyloid process. The dissection completed, the wound was washed out with kreosote solution, and the flaps brought together and secured by the interrupted suture covered with a fold of patent lint, and surmounted by a plaster of simple cerate, then a compress of raw cotton to fill the vacancy and keep the integuments in juxta position with the parts underneath secured with appropriate bandages, and put him to bed and gave him a little wine and water occasionally. During the operation and afterwards, we gave him paregoric, hartshorn, and spirits of cinnamon. In three hours he recovered from exhaustion, and reaction took place with considerable fever and some hemorrhage. I allayed his fever with cold water, and arrested the hemorrhage by kreosote solution and a compress. In three days he could walk about, being free from pain, and able to eat and talk. I left him in charge of Doctors Davidson and Hall. Hemorrhage took place pretty freely on or about the eighth day, from rubbing the wound, and probably tearing away some ligature, but this was even of benefit, as it prevented or restrained all inflammation.

I visited him five weeks afterwards, and found him able to walk about the yard; with good appetite and spirits, and the wound quite healed up. There is quite a chasm instead of an under jaw. After all the blood was washed out of the tumor it weighed three-quarters of a pound. Query, would a strict depleting course and antiphlogistic treatment with a seaton drawn through the tumor at an earlier period, or taking up the inferior maxillary artery, have succeeded in arresting the pain and growth of the tumor?

---

Compound and Comminuted fracture, running into the joint, of the upper part of the Ulna; removal of several inches of the bone; recovery. By Dr. S. M. Meek *of Tuscaloosa, Alabama*, March 1838.

A negro boy, 10 years old, while driving the horse in the cotton mill of his master B. W. had his arm caught and crushed between two cog-wheels. When I arrived two or three hours after the accident, I found the arm in a condition that suggested to the bystanders the necessity of immediate amputation.

The *Supinator iongus, the Pronator radil, Brachialis internus, the Ulnaris externus* and other muscles with the integuments were contused and lacerated, and the upper portion of the ulna and the olecranon were literally mashed to pieces, and the part forming the elbow-joint, separated from the body of the bone. The radius appeared to be but little injured, not broken, but the capsular ligament torn, so that the synovia was discharged, the forearm hanging almost pendulous. After deliberation, I determined to attempt saving the arm. I laid open the integuments, &c., so as to reach the sound part of the ulna, sawed off the fractured fragments, say, between three and four inches, removed the part forming the joint and the olecranon, and dressed by means of ligatures and bandages. Considerable sloughing occurred but the wound soon put on a healthy appearance, and healed kindly, so that in about 10 weeks it was nearly well. In two or three months longer he could use his arm, and at this time can work with axe, hoe, &c. almost as well as any other boy of his size.—The accident happened in December, 1836. At present, 15 months afterwards, he shows but little infirmity.

---

**Peritoneal Enteritis, Schirrus of the Colon, Tubercles of the abdominal and Thoracic Viscera—Death—Dissection.** By W. J. Barbee, *of Indianapolis, now of Crawfordsville, Indiana*, May 1838.

J. G. B., age 52.—Nervo-Sanguineous temperament, enfeebled constitution, sedentary habits of life, predisposed to phthisis, was attacked on Wednesday 11th of April with an acute pain about the first lumbar vertebra extending into both lumbar regions.

For several weeks he had complained of a slightly painful sensation about the kidneys, and labored under some retention of urine, with ardor urinæ. The pain now extended across the abdomen, was intermittent and spasmodic, relieved but slightly by pressure. Some tenderness of stomach, pulse* 80, full and compressible. Tongue has a thin white coat. Bowels obstinately constipated; liver, as for years past, appears to be extremely torpid.

I commenced the treatment of this case by the application of cups over the two or three upper lumbar vertebræ and the administration of a dose of pretty active cathartic pills, ordering fomentations to the abdomen in case of a continuation or increase of pain.

Thursday—5 o'clock A. M.—Medicine has had no effect upon bowels, patient has vomited several times during the past night, but ejects nothing but a colorless, insipid fluid. Pulse 84, somewhat corded, tongue and mouth dryer.

I was about administering a small portion of denarcotized laudanum, but having learned from the patient that the weakest dilution, or any preparation of opium hitherto tried, would make

*Mr. B's. natural pulse was 60.

him delirious, I desisted, and gave him nothing but warm gruel, with a hope that catharsis would follow in a short time.

Stomach was quieted for a few minutes, and the patient was entirely easy, but before a half hour vomiting recommenced and the gruel was thrown up. Siedlitz powders were next given, but to no effect. Oil and turpentine were then administered, and remained on the stomach nearly two hours, finally ejected, at least a good portion thereof. A mustard poultice was applied to the epigastric region and the patient was entirely quiet for an hour.

3 o'clock P, M.—Injections, no effect. Ordered a blister, but previous to its application requested a consultation with Dr. Sanders and Mothershead (my former preceptors, and gentlemen of very respectable standing in the medical profession.) Upon consultation, the warm bath was ordered, blister applied to the stomach, and calomel in ten grain doses repeated every four hours.

Friday—7 o'clock, A. M.—Blister has drawn well, no motion of the bowels, vomiting allayed in some degree, pain in the bowels, pulse 80, tongue dry, edges red—continue calomel and kept up discharges from blister.

3 o'clock, P. M.—Several other physicians called in consultation, determined upon trying the efficacy of an effervescing injection, but this had no other effect than to bring away a few small sciballæ. Drs. S. and M. continued in attendance with myself.

7 o'clock, P. M.—Pulse 85, tense, no vomiting, tongue dry and redder than in the morning, bowels tumefied and painful, pressure upon them has little or no effect. V. S. 16 oz. pulse became softer, fomentations to abdomen.

11 o'clock, P. M.—Croton oil 4 drops in pills, one every hour, no effect; injections of Sol. Tart. Antim. bowels became softer and patient easy, slept two hours.

Saturday—6 o'clock, A. M.—Senna and salts, two doses, ejected by vomiting.

2 o'clock, P. M.—Introduced a gum elastic tube fifteen inches up the colon, and injected a quart of warm water. This produced two or three small fœcal discharges, and bowels became softer and less tumefied.

7. o'clock, P. M.—Blister 10 by 12 inches to the abdomen, mucilaginous drinks, Ol. Ricini to be taken at 10 o'clock.

Sunday—8 o'clock A. M.—Small discharge of black fœces of consistence of thick mush. Blister drew well, kept up discharge, one or two injections by the tube, during the day.

4 o'clock, P. M.—Improving, bland drinks.

Monday—4 o'clock A. M.—A good, large bilious discharge from bowels.

From this time the patient convalesced, and enjoyed a fine prospect of recovery until the succeeding Sunday, at which time he relapsed, and all the former symptoms made their appearance. He was bled, cupped and blistered on the abdomen, and took large and repeated doses of calomel, together with nauseants and other febrifuge medicines. Notwithstanding these means the abdomen continued to dilate till it became quite tympanitic. Gas could be distinctly heard passing along the track of the intestines, and the patient complained of severe intermittent pains, which appeared to be confined chiefly about the umbilicus.

Injections of sugar, salt, warm milk and water, and an emulsion of assafœtida were freely administered by me out of a large flexible tube eighteen inches in length. These seemed to palliate the symptoms considerably and procure sleep for a few hours, but in a short time the various symptoms reappeared in all their violence, and finally became greatly aggravated. The Veratria ointment was applied to the abdomen and Veratria pills were administered, but to no effect. As a dernier resort the trocar was introduced two inches below the umbilicus, and a considerable amount of gas, a few ounces of purulent, bloody serum, together with a small quantity of fœces were discharged. In a few minutes the patient complained of most excruciating pain in the penis which shortly extended itself to the bowels and then became concentrated. A cold clammy sweat broke out upon the surface of the body, the pulse became weak, respiration labored, and in ten hours from the time of the operation he expired.

*Autopsy.—Fifteen hours after death.*

*External appearance.* General and great globular distension of the anterior parietes of the abdomen, no unusual general emaciation.

*Internal appearance. Cavity of lhe abdomen.* Stomach distended; peritoneal and muscular coats healthy; mucous coat inflamed in several patches; omentum two-thirds obliterated. Intestines, with the exception of the descending portion of the colon and rectum, enormously distended by gas, widely diffused hyperæmia of the peritoneal tunic; adhesions to the posterior walls of the abdomen; extensive effusion of coagulated lymph and purulent serum; tubercles studding the descending portion of colon, parts of the mesocolon and rectum, the latter bowel somewhat contracted; mucous coat of the intestines healthy. At a point in the colon about twelve inches from its junction with the rectum a stricture was found, formed by a schirrous tumour, growing from the sub-peritoneal cellular coat inwards, which left a caliber of about a quarter of an inch.

The orifice made by the trocar was found in the ileum not far from its junctian with the cœcum.

*Liver.* Bluish tinge externally—harder than natural, several small tubercles on its surface, otherwise healthy.

*Spleen.* Contracted to a third of its natural size, and full of tubercles.

*Kidneys.* Right healthy, left inflamed, small quantity of pus flowed internally, left rather smaller than natural.

*Bladder.* Healthy, half filled with urine.

*Cavity of the Thorax.* Lungs, with several spots of inflammatory hyperæmia, a number of tubercles, varying in size, from a pinshead to a hazlenut, found in both lungs.

*Heart.* Some hypertrophy of the walls of the left ventricle with contraction of its cavity, an ounce of serum in the pericardium. The brain was not examined.

### Remarks by the author.

It would be rather difficult to apply to the foregoing case a *single name* which would clearly indicate the existence of all the lesions discovered upon post-mortem examination, but I have chosen the appellation of peritoneal enteritis, from the fact, that the peritoneal coat of the bowels, was universally in a state of inflammation. The case throughout, was rather a singular one.

*Pressure on the abdominal parietes, produced no increase of pain, and the pain was intermittent.* Such phenomena are certainly unusual in a lesion of this kind. I did not observe the patient's knees drawn up more than once or twice, throughout the attack, and as will be seen by the record, at times he seemed perfectly free from pain.

Whence came the large quantity of gas contained in the bowels? The patient took very little aliment. I think we are fully justified in the conclusion that it was secreted.

The tumor of the colon was minutely examined, and had all the characteristics of schirrus. Three of the patient's immediate family have died of phthisis.

---

Hernia treated successfully with Dr. Chase's apparatus. By. Dr. Thomas J. Orr, *of Cincinnati.*

In reporting the following cases of Hernia that have been *radically cured* by the use of Dr. Chase's instruments,—I shall not attempt to give the progressive effects of the treatment in each particular instance, but merely the general state of the patient; the condition of the parts concerned when the instrument was applied, and the ultimate results. There could be much said of great practical interest, in relation to the *modus operandi* of these valuable instruments; of the surgical skill, and experience requisite to apply and manage them with success;—the philosophical principles of the cure &c. &c., but this I shall reserve for a paper, when I have had more extended opportunities of observing their effects.

*Case* 1st. Master Henry, aged eight years, a finely formed lad, with a vigorous constitution; has inguinal hernia of the right side, of four years standing;- cause of the accident unknown: external ring large and relaxed; tumor very large and tender to the touch.

June 22d 1836, saw the patient for the first time in company with my friend Dr. Crittenden, who was so kind as to reduce the bowel and apply the common inguinal truss. June 26th, visited the patient, and found that the tumor had not descended. October 10th, saw the boy, and was told by his mother that the retention had been perfect since the time of the first application; removed the instrument and compared the ring with the opposite; found that it had contracted to about the same size, but was firmer, and offered more resistance to the fingers; the instrument remained off for several hours, without the slightest tendency to descent of the tumor; but as the boy was about to start with his parents to Philadelphia, I advised the use of the truss for two months longer; at the expiration of which time it was laid aside. Six months after he returned, and on examination I ascertained that the sac was obliterated at the neck; was filled with fluid and would flow under the finger. I gave two or three brisk cathartics which carried away the water; the patient remains perfectly well to the present date.

*Case* 2d. Inguinal hernia of the right side. Mr. ——— aged thirty, (by profession a pilot,) good constitution, had inguinal hernia for three years, caused by the descent of the testicle of the right side, which protruded partly through the external ring, followed by the hernial tumor which filled the entire Inguinal canal. The testicle could be returned with the tumor into the abdomen. March 20th 1837, forced the testicle through the internal ring, returned the bowel and applied Chase's common Inguinal pad. July 10th, saw the patient, the testicle had gravitated to its proper place in the scrotum along side of its fellow; removed the instrument, found the tumor did not descend, but as there was some pain and weakness, the instrument was re-applied. April 6th, 1838, removed the instrument, and on examination found that there was not the slightest tendency to descent of the tumor; no pain or sense of weakness in the part, and that there was a condensation of cellular matter about the ring, and apparently coalescence of the folds of the sac, which rendered it much firmer than the opposite one. Since the removal of the instrument, the man has performed a trip as pilot from Cincinnati to New-Orleans, without the least evidence of a return of the disease.

*Case* 3d. Mr. G——, aged thirty-five, a merchant; constitution vitiated by habits of intemperance, had inguinal hernia of the right side, of ten years standing. The first discovery of the protusion, was in the morning, after having spent the night previous in laborious dancing. He immediately applied to a surgeon who treated him for several years with the most approved instruments, but without any other effect, than merely to restrain the intestines from descending into the scrotum. In this condition, we were called to see him December 15th 1836, reduced the bowel, and applied the common inguinal pad, which was worn day and night for four months; at this time, the patient anxious to know whether he was well, took off the instrument, but in two or three hours he felt the pain which is experienced when the tumor is about to descend; and instantly applied the truss, which was not removed for six months longer, when it was finally laid aside.

*Case* 4th. Mr. L——, aged twenty-one, of remarkably healthy constitution; followed the river as an occupation:—has inguinal hernia of the right side, caused by falling with his abdomen across the edge of a flat-boat with so much violence as to force a portion of the intestine into the scrotum. In a few hours symptoms of strangulation supervened, but we were able to reduce the bowel without an operation. Chase's Inguinal pad was applied February 14th 1837, and worn until the following June, when the patient being radically cured, it was laid aside.

### Editorial Note.

Dr. Orr has appended to the foregoing cases, a tabular view of 21 cases, which he has treated, but want of space compels us to omit its publication.

The instruments of Dr. Chase have been so long before the profession, and are so generally known, that it is quite unnecessary to say any thing in commendation of them. The cases reported by Dr. Orr, the agent for the State of Ohio, afford additional proof, if any were wanting, of their value in the treatment of hernial disorders. Several of these cases we have witnessed, and we have no hesitation in affirming, that the cures are as perfect as it is possible for nature, assisted by science and art, to make them. Not only are the patients "healed," but the side originally involved, appears really more perfect, and capable of greater resistance than the opposite one. To bring about these effects much skill we presume is required in the application of the instruments, which, in the generality of cases, should only be made by those who have proper tact and experience. S. D. G.

ENCEPHALIC DISORDER, WITH APHONIA AND IMPAIRED INTELLECT, APPARENTLY FROM WORMS. By Dr. J. S. Malone, *of Athens, Alabama, April* 1838.

On the 14th of January 1838, I was requested to visit Miss S. B. G. aged eight years, who had been seized on the 11th, with high fever and dull headache, itching of the nostrils, and complete aphonia. I found her with hot skin, dry and red tongue and fauces, dilated pupils, insensibility to light and speechlessness. Her pulse was 124 in a minute, and her breathing short and quick.

*Treatment.* Bled her to 16 ounces, and gave an emetic, which brought up a quantity of bile with 4 large lumbricoides. A copious perspiration followed and her pulse came down to 88. She then took 20 grains of calomel, 8 of jalap and 20 drops of the oil of turpentine; followed in 13 hours, by the internal use of castor oil and turpentine, with other cathartics, and injections. Free alvine evacuation at length was effected, the discharges were dark and offensive, and contained three additional lumbricoides. Subsequently she was cupped, blistered, nauseated, and took mercurials. On the 27th, a fortnight from the onset of the disease, her breath indicated a slight salivation, and she seemed to be convalescent. February 9th, she was able to sit up, and thenceforth gained flesh and strength rapidly—27th, she was walking about, and attempting, like a child, to talk, but seemed nearly idiotic.

REMARK.

It is to be regretted that Dr. Malone, who wrote on the 16th of April, did not bring down the history of this case to that date. It may be presumed that his patient continued to recover. He asks in a postcript, whether the impairment of the functions of speech and intellect probably resulted from invermination? We suppose it did, as the Aphonia was one of the first symptoms. D.

